# Synthesis, Structure, and Reactivity of Molybdenum– and Tungsten–Indane Complexes with Tris(pyrazolyl)borate Ligand

**DOI:** 10.3390/molecules29040757

**Published:** 2024-02-06

**Authors:** Masumi Itazaki, Kunihisa Nouichi, Ken-ichiro Ookuma, Toshiyuki Moriuchi, Hiroshi Nakazawa

**Affiliations:** 1 Department of Chemistry, Graduate School of Science, Osaka Metropolitan University, Sumiyoshi-ku, Osaka 558-8585, Japan; moriuchi@omu.ac.jp; 2 Department of Chemistry, Graduate School of Science, Osaka City University, Sumiyoshi-ku, Osaka 558-8585, Japan; nouichi@osaka-cu.ac.jp (K.N.); ookuma@sci.oasaka-cu.ac.jp (K.-i.O.)

**Keywords:** molybdenum, tungsten, indium, tris(pyrazolyl)borate, crystal structure

## Abstract

The reaction of molybdenum complexes with a tris(pyrazolyl)borate ligand (Et_4_N[TpMo(CO)_3_] and Et_4_N[Tp*Mo(CO)_3_] (Tp = hydridotris(pyrazolyl)borate, Tp* = hydridotris(3,5-dimethylpyrazolyl)borate)) and InBr_3_ at a 1:1 molar ratio afforded molybdenum–indane complexes (Et_4_N[TpMo(CO)_3_(InBr_3_)] **1** and Et_4_N[Tp*Mo(CO)_3_(InBr_3_)] **2**). In addition, tungsten–indane complexes, Et_4_N[TpW(CO)_3_(InBr_3_)] **3** and Et_4_N[Tp*W(CO)_3_(InBr_3_)] **4**, were obtained by the reaction of corresponding tungsten complexes. Complex **4** reacted with H_2_O to form the hydrido complex Tp*W(CO)_3_H, in which the W–In bond was cleaved. On the other hand, **4** reacted with three equiv. of AgNO_3_ to form Et_4_N[Tp*W(CO)_3_{In(ONO_2_)}] **5**, in which three substituents on the In were exchanged while retaining the W–In dative bond. Complexes **1**–**5** were fully characterized using NMR measurements and elemental analyses, and the structures of **1**–**5** and Et_4_N[Tp*W(CO)_3_] were determined via X-ray crystallography. These are the first examples of mononuclear molybdenum– and tungsten–indane complexes with Mo–In and W–In dative bonds.

## 1. Introduction

**Figure 1:**
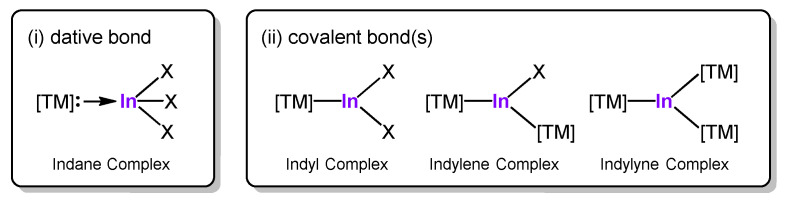
Bond types between TM and indium compound: (**i**) dative bond; (**ii**) covalent bond.

Common compounds with Group 13 elements as the central atom are designated as EX_3_ (E = B, Al, Ga, In, Tl) and are known as electron-deficient compounds. Therefore, for bonds formed between a Group 13 element and a transition metal (TM), a TM→E dative bond can be considered in addition to a TM–E covalent bond (for example, TM–EX_2_) (Figure 1 shows an example in which E = In) [1,2,3,4,5,6,7]. A normal bond between a TM and a ligand is formed by donating two electrons from the ligand to the TM. On the other hand, the TM→E dative bond is a bond created when two electrons are donated from the TM to E. This is called a Z-type interaction and it is a bond that is unique to Group 13 element compounds [8,9,10,11,12,13,14,15]. Although many compounds with TM–B bonds have been reported, there are not many reported examples of compounds with TM–In bonds, and among them, there are few examples of compounds with W–In bonds.

**Figure 2:**
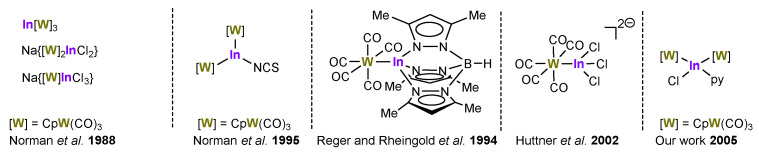
The examples of previously reported W complexes with W–In bond(s) [16,17,18,19,20,21].

Figure 2 shows the complexes with W–In bonds that have been reported to date. In 1988, Norman et al. reported [In{CpW(CO)_3_}_3_], Na[{CpW(CO)_3_}_2_InCl_2_], and Na[{CpW(CO)_3_}InCl_3_] [16] as the first complexes with W–In bond(s). A crystal structure was reported in 2002 for [In{CpW(CO)_3_}_3_] by Scheer [17]. A thiocyanate indylene complex [{CpW(CO)_3_}_2_In(NCS)] was synthesized by Norman in 1995 [18]. Reger and Rheingold used a bulky In compound, Tp*InCl_2_·THF, to prepare the W–In complex [W(CO)_5_(InTp*)] [19]. A trihaloindate W complex [Ph_4_P]_2_[(CO)_5_W–In^I^Cl_3_] containing indium in a formal oxidation state of +I was obtained by the reaction of a reactive In^I^ complex [(CO)_5_WInCl·THF] with an excess amount of [Ph_4_P]Cl [20]. We have also reported the pyridine-stabilized indylene complex [{Cp(CO)_3_W}_2_InCl(py)] [21]. It is reasonable that the W–In bond in the complexes listed above is a covalent bond. However, it is also possible to interpret it as a W→In dative bond. In fact, the nature of the W–In bond is not clearly stated in these papers.

Although there are no examples of structural analyses of complexes with clear W→In dative bonds, several examples of structural analyses of complexes with TM→In dative bonds have been reported for other transition metal complexes: one example for Pt [15], Pd [22], and Ru [23]; two examples for Co [24,25]; and three examples for Ni [26,27,28] and Rh [29,30,31].

In general, polynuclear complexes have several possibilities for how to allocate bonding electrons. The same is true for complexes with TM–In bonds. In other words, when a complex with a TM–In bond has a multinuclear structure, it becomes difficult to determine whether the TM–In bond is a dative bond or a covalent bond. In this study, we focus on complexes with clear TM–In dative bonds. To obtain a complex with a clear TM–In dative bond, it is necessary to prepare a mononuclear complex. Tp (hydridotris(pyrazolyl)borate) and Tp* (hydridotris(3,5-dimethylpyrazolyl)borate) have been widely used as six electron donor anionic ligands, like Cp (cyclopentadienyl) and Cp* (pentamethylcyclopentadienyl) ligands. However, since Tp and Tp* are bulkier than Cp and Cp*, complexes with Tp or Tp* as a ligand are less likely to form polynuclear complexes. Here, we selected Mo and W complexes with a Tp or Tp* ligand and examined their reaction with InBr_3_.

## 2. Results

### 2.1. Synthesis and Structure of Molybdenum–Indane Complexes with Tris(pyrazolyl)borate Ligand

**Scheme 1:**
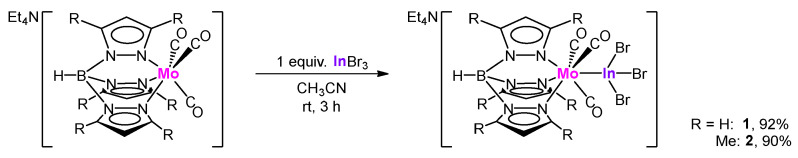
Reactions of Et_4_N[TpMo(CO)_3_] and Et_4_N[Tp*Mo(CO)_3_] with 1 equiv. of InBr_3_.

Our reactions of Et_4_N[TpMo(CO)_3_] [32] and Et_4_N[Tp*Mo(CO)_3_] [32] with one equiv. of InBr_3_ afforded the molybdenum–indane complexes Et_4_N[TpMo(CO)_3_(InBr_3_)] **1** with a 92% yield and Et_4_N[Tp*Mo(CO)_3_(InBr_3_)] **2** with a 90% yield, respectively (Scheme 1). In the ^1^H NMR spectra of **1** and **2** in CD_3_CN, three CH signals (δ 6.30, 7.81, and 8.08) were observed for **1** and one CH signal (δ 5.92) and two CH_3_ signals (δ 2.30 and 2.41) were observed for **2**. However, the BH proton was not observed because of a large nuclear quadrupole moment of B.

**Figure 3:**
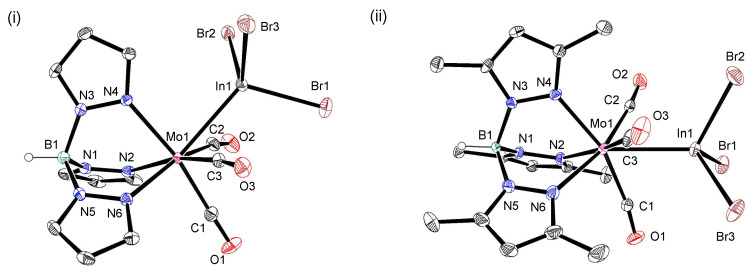
ORTEP drawings of (**i**) the anionic part of Et_4_N[TpMo(CO)_3_(InBr_3_)] **1** and (**ii**) the anionic part of Et_4_N[Tp*Mo(CO)_3_(InBr_3_)] **2** in the crystal (30% thermal ellipsoidal plots). All hydrogen atoms (except for BH proton) are omitted for simplicity.

Single crystals of **1** and **2** were grown from a hot acetonitrile solution. The molecular structures of **1** and **2** were determined via X-ray crystallography. The ethyl groups of cationic parts were disordered, so the cationic parts were omitted for simplicity, and an ORTEP drawing of the anionic parts is depicted in Figure 3 with an atomic numbering scheme. Selected bond lengths (Å) and angles (°) are summarized in Table 1. To estimate the bond interaction of TM–E, the *r* value (the ratio of The M–E bond length to the sum of the TM and E covalent radii) has been used [25,33,34,35]. Thus, the *r* values are also listed in Table 1. Both **1** and **2** are seven-coordinated complexes. The Mo(0) center of **1** has a four-legged piano stool geometry, with one tridentate Tp ligand, three terminal CO ligands, and one InBr_3_ ligand. In contrast, the Mo(0) center of **2** is coordinated by one tridentate Tp* ligand, three terminal CO ligands, and one InBr_3_ ligand in a 3:3:1 face-capped octahedral arrangement. The capped octahedral structure is similar to the previously reported tin analog [Tp*Mo(CO)_3_(SnPh_3_)] [36]. The structural difference between the Tp and Tp* complexes is considered to come from the steric repulsion between the methyl groups on the Tp* ligand and the InBr_3_ ligand. The lengths of the Mo–In bond (2.8575(7) Å for **1**, 2.8117(8) Å for **2**) and In–Br_3_ bond (2.5476(8) Å, 2.5630(9) Å, and 2.5486(9) Å for **1**, 2.5579(9) Å, 2.5408(10) Å, and 2.5467(10) Å for **2**) are shorter than the sum of the Mo and In covalent radii (2.96 Å) and that of the In and Br covalent radii (2.68 Å), respectively [36]. The indium atoms of **1** and **2** have a pseudo-tetrahedral geometry. The Br–In–Br angles are 100.25(2)° to 105.87(3)° for **1** and 102.44(3)° to 103.85(4)° for **2**. To the best of our knowledge, **1** and **2** are the first Mo–indane complexes to be prepared, isolated, and analyzed via their X-ray structure. The Mo–In bond length of **2** is shorter than that of **1**, showing that a greater electron density is donated from Mo to In in **2** than in **1**. The Mo–N bond lengths (an avg. of 2.251 Å for **1** vs. avg. 2.265 Å for Et_4_N[TpMo(CO)_3_] [37], and an 2.238 Å of for **2** vs. avg. 2.263 Å for Et_4_N[Tp*Mo(CO)_3_] [38]) do not change much before and after the coordination of the InBr_3_ ligand to the Mo center. In contrast, a comparison of the Mo–CO bond lengths led to an interesting finding: there was an avg. of 1.977 Å for **1** vs. avg. 1.925 Å for Et_4_N[TpMo(CO)_3_] [37], and an avg. of 1.981 Å for **2** vs. avg. 1.941 Å for Et_4_N[Tp*Mo(CO)_3_] [38]. In both cases of **1** and **2**, the coordination of InBr_3_ to Mo lengthens the Mo–CO bonds. It is thought that the electron density of Mo decreases due to the coordination of the InBr_3_ ligand, weakening the π back-donation to the carbonyl ligands.

### 2.2. Synthesis and Structure of Tungsten–Indane Complexes with Tris(pyrazolyl)borate Ligand

**Scheme 2:**
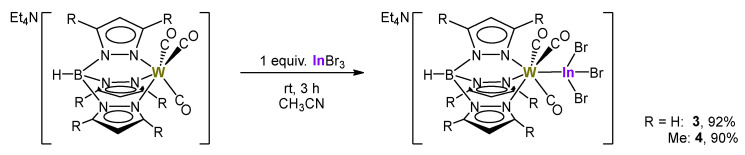
Reactions of Et_4_N[TpW(CO)_3_] and Et_4_N[Tp*W(CO)_3_] with 1 equiv. of InBr_3_.

Tungsten–indane complexes **3** and **4** were obtained with 92% and 90% yields by the reactions of Et_4_N[TpW(CO)_3_] [32] and Et_4_N[Tp*W(CO)_3_] [32] with one equiv. of InBr_3_, respectively (Scheme 2). The ^1^H NMR signals due to the Tp ligand of **3** were observed at δ 6.34, 7.84, and 8.21 in CD_3_CN and those due to the Tp* ligand of **4** were observed at δ 2.35, 2.42, and 5.97 in CD_3_CN. They were slightly downfield compared to the signals of the corresponding Mo–indane complexes (**1** and **2**).

**Figure 4:**
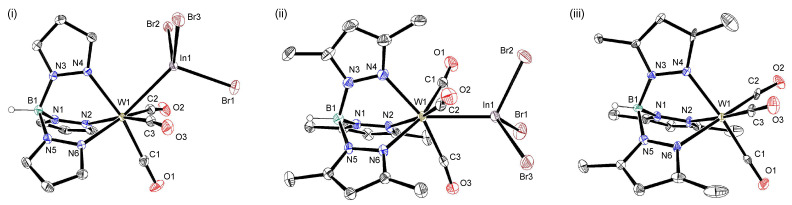
ORTEP drawings of (**i**) the anionic part of Et_4_N[TpW(CO)_3_(InBr_3_)] **3**, (**ii**) the anionic part of Et_4_N[Tp*W(CO)_3_(InBr_3_)] **4**, and (**iii**) the anionic part of Et_4_N[Tp*W(CO)_3_] in the crystal (30% thermal ellipsoidal plots). All hydrogen atoms (except for BH proton) are omitted for simplicity.

Single crystals of **3, 4,** and Et_4_N[Tp*W(CO)_3_] were obtained using the same methods as **1** and **2**, and X-ray crystal analyses were conducted. The ethyl groups of the cationic parts were disordered, so the cationic parts were omitted for simplicity, and ORTEP drawings of the anionic parts are shown in Figure 4. The most characteristic parameters are summarized in Table 2. To the best of our knowledge, **3** and **4** are the first examples of the synthesis and structure of a tungsten–indane complex. The coordination environment around the metal center and the tendencies of the W–N bond lengths and the W–CO bond lengths of **3** and **4** are similar to those of **1** and **2**, respectively. Although the Br–In–Br angles for **3** (99.87(2)° to 105.50(2)°) and **4** (102.63(4)° to 103.41(4)°) are similar to the corresponding **1** and **2**, the *r* values of **3** (0.94) and **4** (0.93) are smaller than those of **1** (0.97) and **2** (0.95), suggesting that the M→In interaction is larger for W than Mo and for the Tp* complex than for the Tp complex. In other words, it is inferred that **4** has the strongest TM→In^III^ dative bond among **1**–**4**.

### 2.3. Reactivity of Tungsten–Indane Complexes with Tp* Ligand

For indane complexes **1**–**4**, which were prepared and isolated in this work, it was shown that the W–In dative bond of **4** was the strongest. Therefore, in order to investigate the possibility of this bond cleavage, the reaction of **4** with a Lewis base was conducted. In addition, in order to investigate whether the In–Br bond can be cleaved while retaining the W–In bond, the reactions of **4** with some Ag salts were examined.

**Scheme 3:**
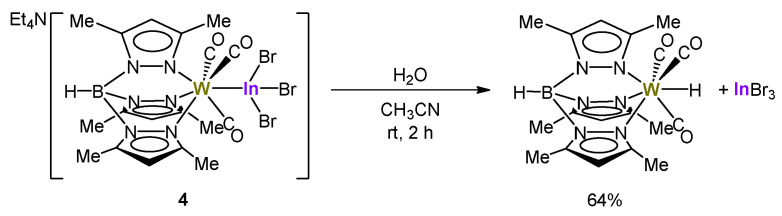
Reaction of Et_4_N[Tp*W(CO)_3_] with H_2_O in acetonitrile.

Water was selected as a Lewis base to react with **4**. Complex **4** was added to water and stirred at room temperature for 2 h, but no change occurred. However, when acetonitrile was added to this, **4** was converted into the hydrido complex (Tp*W(CO)_3_H) with a 64% yield (Scheme 3). Templeton et al. reported that the hydrido complex (Tp*W (CO)_3_H) was obtained in the reaction of Et_4_N[Tp*W(CO)_3_] with HCl [39]. Therefore, our observation shown in Scheme 3 can be explained in the following two ways. (i) InBr_3_ (a Lewis acid) forms a stronger bond with the Tp*W(CO)_3_ fragment than with H_2_O, so InBr_3_ does not dissociate from the W fragment in water, leading to no reaction. On the other hand, InBr_3_ forms a stronger bond with MeCN than Tp*W(CO)_3_, so when MeCN is added, **4** becomes [Tp*W(CO)_3_]^−^ and MeCN–InBr_3_, and the generated [Tp*W(CO)_3_]^−^ reacts with H_2_O to form Tp*W(CO)_3_H. (ii) Since [NEt_4_][Tp*W(CO)_3_(InBr_3_)] (**4**) is poorly soluble in water, no solid–liquid reaction occurred. To investigate the possibility of (ii), we synthesized K[Tp*W(CO)_3_(InBr_3_)], which shows better solubility in water than the NEt_4_ salt, and stirred this complex in water, confirming the formation of Tp*W(CO)_3_H. Therefore, the reason why **4** did not react with water is because of its extremely low solubility in water. The reactions mentioned above revealed that InBr_3_ inherently prefers to interact with H_2_O and MeCN rather than the Tp*W(CO)_3_ fragment.

**Scheme 4:**
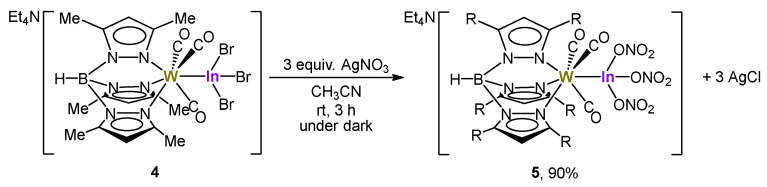
Reaction of Et_4_N[Tp*W(CO)_3_] with 3 equiv. of AgNO_3_.

Since Ag salts are widely used as halogen abstraction reagents, reactions of **4** with some Ag salts were examined. Complex **4** and three equiv. of Ag salt were stirred in MeCN at room temperature for 3 h in the dark. When AgClO_3_, AgOAc, AgOTf, and AgBF_4_ were used, complicated reactions occurred, and the product could not be identified. In the reaction of **4** with AgNO_3_, all Br atoms on the indium were replaced by three NO_3_ groups without breaking the W–In dative bond, and the corresponding complex Et_4_N[Tp*W(CO)_3_{In(ONO_2_)}] (**5**) was obtained with a 90% yield (Scheme 4). This method seems to be applicable to the synthesis of various transition metal–indane complexes.

**Figure 5:**
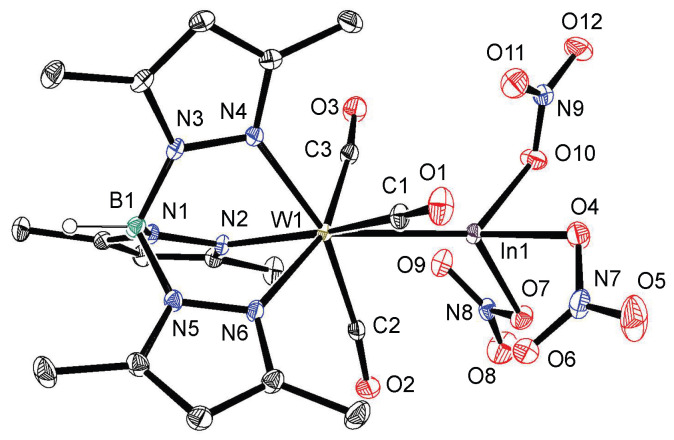
ORTEP drawing of anionic part of Et_4_N[Tp*W(CO)_3_{In(ONO_2_)_3_}] **5**·CH_3_CN in the crystal (30% thermal ellipsoidal plots). The solvent for the crystallization (CH_3_CN), as well as all hydrogen atoms (except for BH proton) are omitted for simplicity.

Complex **5** was characterized via a single-crystal X-ray diffraction analysis. The cationic part was omitted for simplicity, an ORTEP drawing of the anionic part is shown in Figure 5, and selected bond lengths, angles, and *r* values are summarized in Table 3. The geometry about the W atom is a capped octahedral with the indium located at an axial position and a structure that is analogous to that of **4**. Since In has a high electron-accepting ability, when it has an NO_3_ substituent, it often adopts an η^2^-type bonding mode and accepts three electrons rather than adopting an η^1^-type bonding mode and accepting one electron. The crystal structure of an In compound with three NO_3_ groups [In(4,4′-dmbipy)(η^2^-NO_3_)_2_(η^1^-NO_3_)(H_2_O)] (4,4′-dmbipy = 4,4′-dimethyl-2,2′-bipyridine) was reported, in which two NO_3_ are bonded in the η^2^-type bonding mode and one NO_3_ is bonded in the η^1^-type bonding mode to the In (η^2^-type In–O bond lengths: 2.291(12)–2.546(11) Å; η^1^-type In–O bond length: 2.203(8) Å) [40]. In the crystal structure of **5**, in any three NO_3_ groups, one In–O length (2.176(2)–2.183(2) Å) is significantly shorter than the other two In–O lengths (2.623–2.744 Å). Thus, all of the three NO_3_ groups in the In form η^1^-type bonds (only one O atom makes a bond with In), and none of them form η^2^-type bonds (base-stabilized type). This can be considered to be a reflection of the Tp*W(CO)_3_ fragment donating a sufficient electron density to the In. Its *r* value of **5** (0.92) is the smallest among those of the complexes reported in this work.

## 3. Materials and Methods

### 3.1. General Considerations

All manipulations were carried out using standard Schlenk techniques under a dry nitrogen atmosphere. Molybdenum and tungsten complexes with Tp or Tp* ligands (Et_4_N[TpM(CO)_3_] and Et_4_N[Tp*M(CO)_3_] (M = Mo, W)) were prepared according to a method from the literature [32]. The other chemicals were commercially available. Solvents were purified employing a two-column solid-state purification system or were distilled from appropriate drying agents under N_2_. NMR spectra (^1^H and ^13^C) were recorded at ambient temperature on a JEOL JNM AL-400 spectrometer. ^1^H and ^13^C NMR data referred to residual peaks of solvent as an internal standard. Elemental analysis data were obtained with a Perkin–Elmer 2400 CHN elemental analyzer.

### 3.2. Syntheses

Et_4_N[TpMo(CO)_3_(InBr_3_)] (**1**). An acetonitrile solution (35 mL) containing Et_4_N[TpMo(CO)_3_] (310.1 mg, 0.59 mmol) and InBr_3_ (212.1 mg, 0.60 mmol) was stirred at room temperature. After 3 h, all volatile materials were removed under reduced pressure. The residual powder was washed with Et_2_O (15 mL × 4) and dried in vacuo to obtain **1** (478.0 mg, 92%) as a yellow-green powder. ^1^H NMR (400 MHz, CD_3_CN, ppm) spectra were as follows: δ 1.18 (m, 12H, CH_2_CH_3_), 3.14 (q, *J*_HH_ = 7.2 Hz, 8H, CH_2_CH_3_), 6.30 (t, *J*_HH_ = 2.4 Hz, 3H, 4-H), 7.81 (d, *J*_HH_ = 2.4 Hz, 3H, 5-H), 8.08 (d, *J*_HH_ = 2.4 Hz, 3H, 3-H). ^13^C NMR (100.4 MHz, CD_3_CN, ppm) spectra were as follows: δ 7.76 (s, CH_2_CH_3_), 53.05 (m, CH_2_CH_3_), 107.45 (s, 4-C), 137.65 (s, 5-C), 146.89 (s, 3-C), 223.94 (s, CO). Elemental analysis (%) calcd for C_20_H_30_BBr_3_N_7_O_3_InMo was as follows: C, 27.37; H, 3.44; N, 11.17. Found: C, 27.28; H, 3.35; N, 10.89%.

Et_4_N[Tp*Mo(CO)_3_(InBr_3_)] (**2**). In a procedure analogous to the outline above, Et_4_N[Tp*Mo(CO)_3_] (407.2 mg, 0.67 mmol) and InBr_3_ (244.6 mg, 0.69 mmol) obtained **2** (578.1 mg, 0.60 mmol, 90%) as a yellow powder. ^1^H NMR (400 MHz, CD_3_CN, ppm) spectra were as follows: δ 1.20 (m, 12H, CH_2_CH_3_), 2.30 (s, 9H, 5-Me), 2.41 (s, 9H, 3-Me), 3.14 (q, *J*_HH_ = 7.2 Hz, 8H, CH_2_CH_3_), 5.92 (s, 3H, 4-H). ^13^C NMR (100.4 MHz, CD_3_CN, ppm) spectra were as follows: δ 7.71 (s, CH_2_CH_3_), 12.99 (s, 5-Me), 15.68 (s, 3-Me), 53.02 (s, CH_2_CH_3_), 107.73 (s, 4-C), 146.92 (s, 5-C), 152.69 (s, 3-C), 229.39 (s, CO). Elemental analysis (%) calcd for C_26_H_42_BBr_3_N_7_O_3_InMo was as follows: C, 32.46; H, 4.40; N, 10.19. Found: C, 32.54; H, 4.34; N, 10.04%.

Et_4_N[TpW(CO)_3_(InBr_3_)] (**3**). In a procedure analogous to the outline above, Et_4_N[TpW(CO)_3_] (359.4 mg, 0.59 mmol) and InBr_3_ (211.3 mg, 0.60 mmol) obtained **3** (567.9 mg, 0.59 mmol, 92%) as a yellow powder. ^1^H NMR (400 MHz, CD_3_CN, ppm) spectra were as follows: δ 1.13 (br, 12H, CH_2_CH_3_), 3.16 (q, *J*_HH_ = 7.2 Hz, 8H, CH_2_CH_3_), 6.34 (s, 3H, 4-H), 7.84 (s, 3H, 5-H), 8.21 (s, 3H, 3-H). ^13^C NMR (100.4 MHz, CD_3_CN, ppm) spectra were as follows: δ 7.76 (s, CH_2_CH_3_), 53.05 (m, CH_2_CH_3_), 107.45 (s, 4-C), 137.65 (s, 5-C), 146.89 (s, 3-C), 223.94 (s, CO). Elemental analysis (%) calcd for C_20_H_30_BBr_3_N_7_O_3_InW was as follows: C, 24.88; H, 3.13; N, 10.15. Found: C, 24.61; H, 3.08; N, 9.78%.

Et_4_N[Tp*W(CO)_3_(InBr_3_)] (**4**). In a procedure analogous to the outline above, Et_4_N[Tp*W(CO)_3_] (134.4 mg, 0.19 mmol) and InBr_3_ (74.1 mg, 0.21 mmol) obtained **4** (183.2 mg, 0.18 mmol, 90%) as a yellow-green powder. ^1^H NMR (400 MHz, CD_3_CN, ppm) spectra were as follows: δ 1.20 (br, 12H, CH_2_CH_3_), 2.35 (s, 9H, 5-Me), 2.42 (s, 9H, 3-Me), 3.15 (q, *J*_HH_ = 7.2 Hz, 8H, CH_2_CH_3_), 5.97 (s, 3H, 4-CH. ^13^C NMR (100.4 MHz, CD_3_CN, ppm) spectra were as follows: δ 5.90 (s, CH_2_CH_3_), 11.11 (s, 5-Me), 14.44 (s, 3-Me), 51.22 (m, CH_2_CH_3_), 106.14 (s, 4-C), 145.00 (s, 5-C), 151.05 (s, 3-C), 224.12 (s, CO). Elemental analysis (%) calcd for C_26_H_42_BBr_3_N_7_O_3_InW was as follows: C, 29.75; H, 4.03; N, 9.34. Found: C, 29.60; H, 3.96; N, 9.09%.

Et_4_N[Tp*W(CO)_3_{In(ONO_2_)_3_}] (**5**). An acetonitrile solution (30 mL) containing **4** (114.7 mg, 0.11 mmol) and AgNO_3_ (55.8 mg, 0.33 mmol) was stirred at room temperature under dark conditions. After 3 h, the formed solid product was collected by filtration and dried in vacuo to obtain **5** (98.1 mg, 0.10 mmol, 90%) as a dark-yellow powder. ^1^H NMR (400 MHz, CD_3_CN, ppm) spectra were as follows: δ 1.19 (br, 12H, CH_2_CH_3_), 2.28 (s, 9H, 5-Me), 2.43 (s, 9H, 3-Me), 3.14 (br, 8H, CH_2_CH_3_), 6.00 (s, 3H, 4-H). ^13^C NMR (100.4 MHz, CD_3_CN, ppm) spectra were as follows: δ 7.70 (s, CH_2_CH_3_), 13.02 (s, 5-Me), 15.92 (s, 3-Me), 53.03 (m, CH_2_CH_3_), 108.47 (s, 4-C), 147.45 (s, 5-C), 153.20 (s, 3-C), 224.41 (s, CO). Elemental analysis (%) calcd for C_26_H_42_BN_10_O_12_InW was as follows: C, 31.35; H, 4.25; N, 14.06. Found: C, 31.67; H, 4.25; N, 14.48%.

### 3.3. Crystallography

Crystallographic data and details of structure refinement parameters are summarized in Table 4 and Table 5. The single crystals **1**–**4** were grown from a hot acetonitrile solution. The single crystals of **5** were obtained using the slow diffusion method (acetonitrile/ether). Diffraction intensity data were collected with a Rigaku AFC11 with Saturn 724+ CCD diffractometer, and a semiempirical multi-scan absorption correction [41] was performed. The structures were solved using SIR97 [42] by subsequent difference Fourier syntheses and refined by full-matrix least-squares procedures on F^2^. All non-hydrogen atoms were refined with anisotropic displacement coefficients. Hydrogen atoms were treated as idealized contributions and refined in rigid group model. All software and sources of scattering factors are contained in the SHELXL-97 [43] and the SHELXL-2018/3 [44] program package. The Cambridge Crystallographic Data Centre (CCDC) deposition numbers of **1–5** and Et_4_N[Tp*W(CO)_3_] are included in Table 4 and Table 5.

## 4. Conclusions

We are able to show the first examples of the synthesis and structure of molybdenum– and tungsten–indane complexes (**1**–**4**). These complexes were obtained by the reactions of corresponding molybdenum and tungsten complexes with Tp or Tp* ligands with one equiv. of InBr_3_. The structural difference between the Tp complexes (**1** and **3** having a four-legged piano stool geometry) and the Tp* complexes (**2** and **4** having a 3:3:1 face-capped octahedral geometry) is considered to be derived from the steric repulsion between the methyl groups on the Tp* ligand and the InBr_3_ ligand. The reaction of **4** with H_2_O afforded the hydrido complex Tp*W(CO)_3_H as a result of the W–In bond cleavage. In contrast, the reaction of **4** with three equiv. of AgNO_3_ produced Et_4_N[Tp*W(CO)_3_{In(ONO_2_)}] **5**, in which the In–Br bonds are cleaved while retaining the W–In dative bond. We believe that the findings obtained in this paper will contribute to our knowledge of the chemistry of transition metal–indane complexes.

## Figures and Tables

**Table 1:** Selected bond lengths (Å) and angles (°) for **1** and **2**.

	1	2
Mo1–In1	2.8575(7)	2.8117(8)
*r * ^a^	0.97	0.95
Mo1–C1	1.967(4)	1.978(7)
Mo1–C2	1.977(5)	1.983(7)
Mo1–C3	1.987(4)	1.982(7)
Mo1–N2	2.238(3)	2.230(5)
Mo1–N4	2.295(3)	2.242(5)
Mo1–N6	2.219(3)	2.241(6)
In1–Br1	2.5476(8)	2.5579(9)
In1–Br2	2.5630(9)	2.5408(10)
In1–Br3	2.5486(9)	2.5467(10)
C1–Mo1–In1	107.64(13)	67.68(18)
C2–Mo1–In1	62.52(12)	65.40(18)
C3–Mo1–In1	64.30(12)	66.68(18)

^a^ Ratio of the Mo–In bond length to the sum of Mo and In covalent radii [35].

**Table 2:** Selected bond lengths (Å) and angles (°) for **3**, **4**, and Et_4_N[Tp*W(CO)_3_].

	3	4	Et_4_N[Tp*W(CO)_3_]
W1–In1	2.8663(4)	2.8287(7)	
*r* ^a^	0.94	0.93	
W1–C1	1.968(5)	1.970(9)	1.940(10)
W1–C2	1.975(5)	1.970(8)	1.933(11)
W1–C3	1.979(5)	1.975(8)	1.942(10)
W1–N2	2.229(4)	2.236(6)	2.258(8)
W1–N4	2.280(4)	2.244(6)	2.249(8)
W1–N6	2.206(4)	2.225(6)	2.241(9)
In1–Br1	2.5527(6)	2.5453(11)	
In1–Br2	2.5719(6)	2.5456(11)	
In1–Br3	2.5554(7)	2.5608(11)	
C1–W1–In1	107.77(16)	66.3(2)	
C2–W1–In1	62.53(15)	67.3(2)	
C3–W1–In1	64.50(15)	65.2(2)	

^a^ Ratio of the W–In bond length to the sum of W and In covalent radii [35].

**Table 3:** Selected bond lengths (Å) and angles (°) for **5**.

	**5**·CH_3_CN
W1–In1	2.7862(4)
*r* ^a^	0.92
W1–C1	1.980(3)
W1–C2	1.984(3)
W1–C3	1.990(3)
W1–N2	2.234(2)
W1–N4	2.235(2)
W1–N6	2.238(2)
In1–O4	2.183(2)
In1–O7	2.176(2)
In1–O10	2.180(2)
C1–W1–In1	67.32(8)
C2–W1–In1	66.38(8)
C3–W1–In1	66.26(9)

^a^ Ratio of the W–In bond length to the sum of W and In covalent radii [35].

**Table 4:** Crystallographic data and details of structure refinement parameters of **1**, **2**, and Et_4_N[Tp*W(CO)_3_].

	1	2	Et_4_N[Tp*W(CO)_3_]
empirical formula	C_20_H_30_BBr_3_N_7_O_3_InMo	C_26_H_42_BBr_3_N_7_O_3_InMo	C_26_H_42_BN_7_O_3_W
formula weight	877.81	961.97	695.32
*T* (K)	200(2)	200(2)	200(2)
crystal system	monoclinic	monoclinic	orthorhombic
space group	*P*2_1_/*c*	*P*2_1_/*c*	*Pnma*
*a* (Å)	9.752(3)	10.7918(15)	17.9483(19)
*b* (Å)	20.721(6)	18.956(2)	16.7315(16)
*c* (Å)	15.341(5)	18.011(2)	9.8651(11)
*β* (°)	102.664(4)	103.763(3)	
volume (Å^3^)	3024.5(16)	3578.8(8)	2962.5(5)
Z	4	4	4
*ρ*_calcd_ (mg m^−3^)	1.928	1.785	1.559
*μ* (mm^−1^)	5.174	4.381	3.938
*F*(000)	1696	1888	1400
crystal size (mm^3^)	0.32 × 0.15 × 0.05	0.20 × 0.12 × 0.07	0.45 × 0.06 × 0.05
reflections collected	24,066	34,788	29,203
R(int)	6890 (0.0375)	8146 (0.0494)	4030 (0.0437)
*R*1 (*I* > 2σ(*I*)) ^a^	0.0414	0.0661	0.0352
w*R*2 (all data) ^b^	0.0772	0.1205	0.0919
goodness of fit	1.000	1.036	1.247
CCDC deposition number	2,320,433	2,320,436	2,321,243

^a^ *R*1 = ∑||*F*_o_| − |*F*_c_||/∑|*F*_o_|. ^b^ w*R*2 = [∑w(*F*^2^_o_ − *F*^2^_c_)^2^/∑w(*F*^2^_o_)^2^]^1/2^.

**Table 5:** Crystallographic data and details of structure refinement parameters of **3**–**5**.

	3	4	5·CH_3_CN
empirical formula	C_20_H_30_BBr_3_N_7_O_3_InW	C_26_H_42_BBr_3_N_7_O_3_InW	C_28_H_45_BN_11_O_12_InW
formula weight	965.72	1049.88	1037.23
*T* (K)	200(2)	203(2)	200(2)
crystal system	monoclinic	monoclinic	monoclinic
space group	*P*2_1_/*c*	*P*2_1_/*n*	*P*2_1_/*n*
*a* (Å)	9.7682(5)	10.7972(14)	10.432(2)
*b* (Å)	20.6886(9)	18.926(2)	18.986(4)
*c* (Å)	15.3167(8)	18.664(3)	19.975(4)
*β* (°)	102.830(2)	110.590(3)	101.117(2)
volume (Å^3^)	3018.1(3)	3570.3(8)	3882.3(13)
Z	4	4	4
*ρ*_calcd_ (mg m^−3^)	2.125	1.953	1.775
*μ* (mm^−1^)	8.578	7.259	3.626
*F*(000)	1824	2016	2056
crystal size (mm^3^)	0.10 × 0.10 × 0.06	0.18 × 0.10 × 0.06	0.32 × 0.10 × 0.10
reflections collected	23,893	35,171	30,360
R(int)	6860 (0.0327)	8138 (0.0511)	8813 (0.0260)
*R*1 (*I* > 2σ(*I*)) ^a^	0.0358	0.0568	0.0263
w*R*2 (all data) ^b^	0.0649	0.1115	0.0526
goodness of fit	1.000	1.001	1.000
CCDC deposition number	2,320,437	2,320,441	2,320,443

^a^ *R*1 = ∑||*F*_o_| − |*F*_c_||/∑|*F*_o_|. ^b^ w*R*2 = [∑w(*F*^2^_o_ − *F*^2^_c_)^2^/∑w(*F*^2^_o_)^2^]^1/2^.

## Data Availability

The crystallographic data are available from the Cambridge Crystallographic Data Centre (CCDC). Other data not presented in Supplementary Materials are available upon request from the corresponding author.

## Supplementary Materials

The following supporting information can be downloaded at: https://www.mdpi.com/article/10.3390/molecules29040757/s1. Figures S1–S10: NMR spectra of all new compounds, Figures S11–S16: Crystal packings of **1**–**5** and Et_4_N[Tp*W(CO)_3_].

## References

[1] Shriver D.F. (1970). Transition metal basicity. Acc. Chem. Res..

[2] Baker R.J., Jones C. (2005). The coordination chemistry and reactivity of group 13 metal(I) heterocycles. Coord. Chem. Rev..

[3] Asay M., Jones C., Driess M. (2011). N-Heterocyclic Carbene Analogues with Low-Valent Group 13 and Group 14 Elements: Syntheses, Structures, and Reactivities of a New Generation of Multitalented Ligands. Chem. Rev..

[4] González-Gallardo S., Bollermann T., Fischer R.A., Murugavel R. (2012). Cyclopentadiene Based Low-Valent Group 13 Metal Compounds: Ligands in Coordination Chemistry and Link between Metal Rich Molecules and Intermetallic Materials. Chem. Rev..

[5] Bouhadir G., Bourissou D. (2016). Complexes of ambiphilic ligands: Reactivity and catalytic applications. Chem. Soc. Rev..

[6] Abdalla J.A.B., Aldridge S., Liddle S.T. (2015). Molecular Metal-Metal Bonds: Compounds, Synthesis Properties.

[7] Bouhadir G., Amgoune A., Bourissou D., Hill A.F., Fink M.J. (2010). Chapter 1 phosphine-boranes and related ambiphilic compounds: Synthesis, structure, and coordination to transition metals. Advances in Organometallic Chemstry.

[8] Cowie B.E., Emslie D.J.H. (2014). Platinum Complexes of a Borane-Appended Analogue of 1,1’-Bis(diphenylphosphino)ferrocene: Flexible Borane Coordination Modes and in situ Vinylborane Formation. Chem. Eur. J..

[9] Barnett B.R., Moore C.E., Rheingold A.L., Figueroa J.S. (2014). Cooperative Transition Metal/Lewis Acid Bond-Activation Reactions by a Bidentate (Boryl)iminomethane Complex: A Significant Metal–Borane Interaction Promoted by a Small Bite-Angle LZ Chelate. J. Am. Chem. Soc..

[10] Cowie B.E., Tsao F.A., Emslie D.J.H. (2015). Synthesis and Platinum Complexes of an Alane-Appended 1,1’-Bis(phosphino)ferrocene Ligand. Angew. Chem. Int. Ed..

[11] Devillard M., Bouhadir G., Bourissou D. (2015). Cooperation between Transition Metals and Lewis Acids: A Way To Activate H_2_ and H–E bonds. Angew. Chem. Int. Ed..

[12] Shih W.C., Gu W.X., MacInnis M.C., Timpa S.D., Bhuvanesh N., Zhou J., Ozerov O.V. (2016). Facile Insertion of Rh and Ir into a Boron–Phenyl Bond, Leading to Boryl/Bis(phosphine) PBP Pincer Complexes. J. Am. Chem. Soc..

[13] Devillard M., Declercq R., Nicolas E., Ehlers A.W., Backs J., Saffon-Merceron N., Bouhadir G., Slootweg J.C., Uhl W. (2016). Bourissou, D.A Significant but Constrained Geometry Pt→Al Interaction: Fixation of CO_2_ and CS_2_, Activation of H_2_ and PhCONH_2_. J. Am. Chem. Soc..

[14] Li Y., Hou C., Jiang J., Zhang Z., Zhao C., Page A.J., Ke Z. (2016). General H_2_ Activation Modes for Lewis Acid–Transition Metal Bifunctional Catalysts. ACS Catal..

[15] Bertermann R., Bönke J., Braunschweig H., Dewhurst R.D., Kupfer T., Muessig J.H., Pentecost L., Radacki K., Sen S.S., Varga A. (2016). Dynamic, Reversible Oxidative Addition of Highly Polar Bonds to a Transition Metal. J. Am. Chem. Soc..

[16] Clarkson L.M., Clegg W., Norman N.C., Tucker A.J., Webster P.M. (1988). Structural studies of organomolybdenum complexes containing indium. Inorg. Chem..

[17] Leiner E., Scheer M. (2002). Cp*GaCl_2_ and Cp*_2_GaCl (Cp* = η^1^-C_5_Me_5_) as starting materials for novel Group 13 element complexes. J. Organomet. Chem..

[18] Carmalt C.J., Norman N.C., Pember R.F., Farrugia L.J. (1995). Synthetic and structural studies on organotransition metal-indium thiocyanate complexes. Polyhedron.

[19] Reger D.L., Mason S.S., Rheingold A.L., Haggerty B.S., Arnold F.P. (1994). Syntheses and Solid State Structures of [HB(3,5-Me_2_pz)_3_]InFe(CO)_4_ and [HB(3,5-Me_2_pz)_3_]InW(CO)_5_ (pz = Pyrazolyl Ring). Intermetallic Complexes with Short Metal-Metal Bonds. Organometallics.

[20] Rutsch P., Renner G., Huttner G., Sandhöfner S. (2002). Organometallically protected indates and germanates. Synthesis, structure and properties of [{(CO)_5_M}EX_3_]^2−^ (M = Cr, Mo, W.; E = In; X = Cl, Br), [(CO_5_Cr)InBr(μ^2^-Br)]_2_^2−^ and [{(CO)_5_Cr}E(oxinate)_2_]^n−^ (E = In, n = 2; E = Ge, n = 1). Z. Naturforsch. B.

[21] Nakazawa H., Itazaki M., Owaribe M. (2005). Chloropyridinebis[tricarbonyl(h^5^-cyclopentadienyl)tungstenio]indium. Acta Crystallogr. Sect. E.

[22] Derrah E.J., Sircoglou M., Mercy M., Ladeira S., Bouhadir G., Miqueu K., Maron L., Bourissou D. (2011). Original Transition Metal→Indium Interactions upon Coordination of a Triphosphine−Indane. Organometallics.

[23] Itazaki M., Ito M., Nakazawa H. (2015). Synthesis, Structure, and Reactivity of Ruthenium(0) Indane Complexes *fac*-[Ru(NCMe)_3_(CO)_2_(InX_3_)] (X = Cl, Br). Eur. J. Inorg. Chem..

[24] Vollmer M.V., Ye J., Linehan J.C., Graziano B.J., Preston A., Wiedner E.S., Lu C.C. (2020). Cobalt-Group 13 Complexes Catalyze CO_2_ Hydrogenation via a Co(−I)/Co(I) Redox Cycle. ACS Catal..

[25] Vollmer M.V., Xie J., Lu C.C. (2017). Stable Dihydrogen Complexes of Cobalt(−I) Suggest an Inverse *trans*-Influence of Lewis Acidic Group 13 Metalloligands. J. Am. Chem. Soc..

[26] Cammarota R.C., Xie J., Burgess S.A., Vollmer M.V., Vogiatzis K.D., Ye J., Linehan J.C., Appel A.M., Hoffmann C., Wang X. (2019). Thermodynamic and kinetic studies of H_2_ and N_2_ binding to bimetallic nickel-group 13 complexes and neutron structure of a Ni(η^2^-H_2_) adduct. Chem. Sci..

[27] Vollmer M.V., Cammarota R.C., Lu C.C. (2019). Reductive Disproportionation of CO_2_ Mediated by Bimetallic Nickelate(–I)/Group 13 Complexes. Eur. J. Inorg. Chem..

[28] Cammarota R.C., Lu C.C. (2015). Tuning Nickel with Lewis Acidic Group 13 Metalloligands for Catalytic Olefin Hydrogenation. J. Am. Chem. Soc..

[29] Woińska M., Pawlędzio S., Chodkiewicz M.L., Woźniak K. (2023). Hirshfeld Atom Refinement of Metal–Organic Complexes: Treatment of Hydrogen Atoms Bonded to Transition Metals. J. Phys. Chem. A.

[30] Steinke T., Gemel C., Cokoja M., Wintera M., Fischer R.A. (2003). Insertion of organoindium carbenoids into rhodium halide bonds: Revisiting a classic type of transition metal–group 13 metal bond formation. Chem. Commun..

[31] Moore J.T., Lu C.C. (2020). Catalytic Hydrogenolysis of Aryl C–F Bonds Using a Bimetallic Rhodium−Indium Complex. J. Am. Chem. Soc..

[32] Trofimenko S. (1969). Transition Metal Polypyrazolylborates Containing Other Ligands. J. Am. Chem. Soc..

[33] Cotton F.A., Murillo C.A. (2007). Multiple Bonds between Metal Atoms.

[34] Amgoune A., Bourissou D. (2011). σ-Acceptor, Z-type ligands for transition metals. Chem. Commun..

[35] Cordero B., Gómez V., Platero-Prats A.E., Revés M., Echeverría J., Cremades E., Barragán F., Alvarez S. (2008). Covalent radii revisited. Dalton Trans..

[36] Liu Y.-Y., Mar A., Rettig S.J., Storr A., Trotter J. (1988). Molybdenum-tin and molybdenum-copper complexes derived from the molybdenum tricarbonyl anions, LMo(CO)^−^ (where L = HBpz_3_ or HBpz”_3_; pz = pyrazolyl, N_2_C_3_H_3_; and pz” = 3,5-dimethylpyrazolyl, N_2_C_5_H_7_). Can. J. Chem..

[37] Shiu K.-B., Lee J.Y., Wang Y., Cheng M.-C., Wang S.-L., Liao F.-L. (1993). Structures and properties of molybdenum carbonyl complexes containing uninegative nitrogen-tripod ligands derived from heterocyclic compounds including 1-H-pyrazole and l-H-1,2,4-triazole. J. Organomet. Chem..

[38] Marabella C.P., Enemark J.H. (1982). The crystal and molecular structure of tetraethylammonium hydrotris(3,5-dimethylpyrazolyl)boratotricarbonylmolybdenum(0), [NEt_4_][Mo(CO)_3_HB(C_5_H_7_N_2_)_3_]. J. Organomet. Chem..

[39] Caffyn A.J.M., Feng S.G., Dierdorf A., Gamble A.S., Eldredge P.A., Vossen M.R., White P.S., Templeton J.L. (1991). Unusual proton NMR properties of tungsten(II) tris(pyrazolyl)borate hydride complexes. Organometallics.

[40] Delbari A.S., Shahvelayati A.S., Jodaian V., Amani V. (2015). Mononuclear and dinuclear indium(III) complexes containing methoxy and hydroxy-bridge groups, nitrate anion and 4,4’-dimethyl-2,2’-bipyridine ligand: Synthesis, characterization, crystal structure determination, luminescent properties, and thermal analyses. J. Iran Chem. Soc..

[41] Rigaku (1998). REQAB.

[42] Altomare A., Burla M.C., Camalli M., Cascarano G., Giacovazzo C., Guagliard A., Moliterni A.G.G., Spagna R. (1999). SIR97: A new tool for crystal structure determination and refinement. J. Appl. Crystallogr..

[43] Sheldrick G.M. (1997). Program for Crystal Structure Refinement.

[44] Sheldrick G.M. (2015). Crystal structure refinement with SHELXL. Acta Crystallogr..

