# Supplementary material for: Synthesis, Structure, and Reactivity of Molybdenum– and Tungsten–Indane Complexes with Tris(pyrazolyl)borate Ligand
Source: Molecules. 2024 Feb 6;29(4):757. doi: 10.3390/molecules29040757 (PMC10893353; doi:10.3390/molecules29040757)
Supplement: Supplementary file 1 [file molecules-29-00757-s001.zip › molecules-2818602-supplementary.pdf]

SUPPORTING INFORMATION

Article

Synthesis, Structure, and Reactivity of Molybdenum– and Tungsten–Indane Complexes with Tris(pyrazolyl)borate Ligand

Masumi Itazaki [1,2,*], Kunihisa Nouichi [2], Ken-ichiro Ookuma [2], Toshiyuki Moriuchi [1,2] and Hiroshi Nakazawa [1,2,*]

1 Department of Chemistry, Graduate School of Science, Osaka Metropolitan University, Sumiyoshi-ku, Osaka 558-8585, Japan; moriuchi@omu.ac.jp
2 Department of Chemistry, Graduate School of Science, Osaka City University, Sumiyoshi-ku, Osaka 558-8585, Japan; nouichi@osaka-cu.ac.jp (K.N.); ookuma@sci.oasaka-cu.ac.jp (K.-i.O.)
* Correspondence: mitazaki@omu.ac.jp (M.I.); nakazawa@omu.ac.jp (H.N.); Tel.: +81-6-6605-3123 (M.I.); +81-6-6605-2547 (H.N.)

Table of Contents | Page

NMR spectra of all new compounds S1–S4

Crystal packing of 1-5 and Et4N[Tp*W(CO)3] S4–S7

NMR spectra of all new compounds

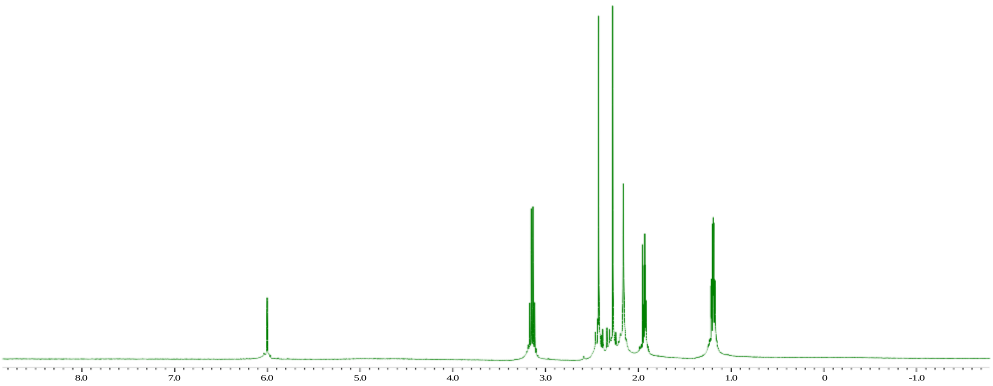

Figure S1. ^{1}H NMR spectrum (400 MHz, CD_3CN, 20 °C) of **1**.

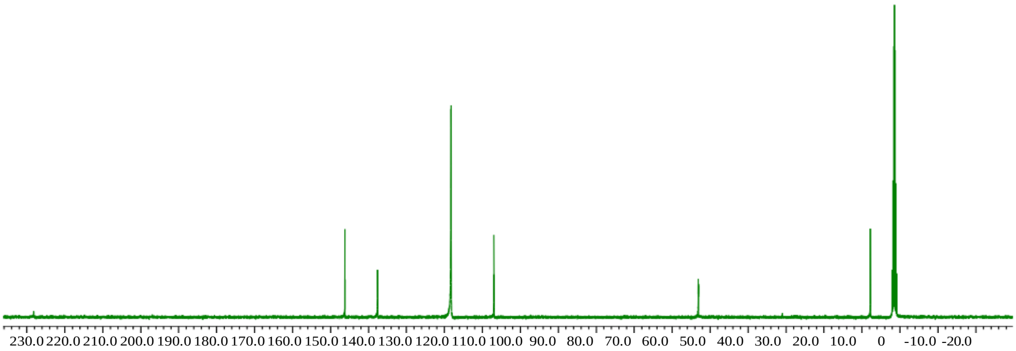

Figure S2. ^{13}C NMR spectrum (100.4 MHz, CD_3CN, 20 °C) of **1**.

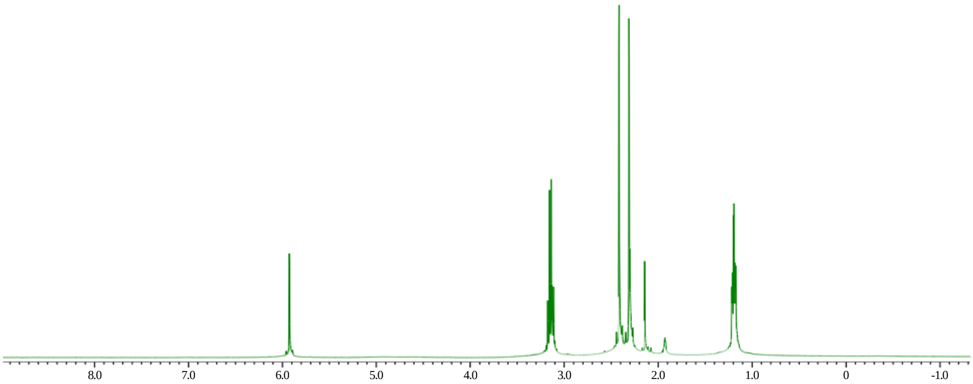

Figure S3. ^{1}H NMR spectrum (400 MHz, CD_3CN, 20 °C) of **2**.

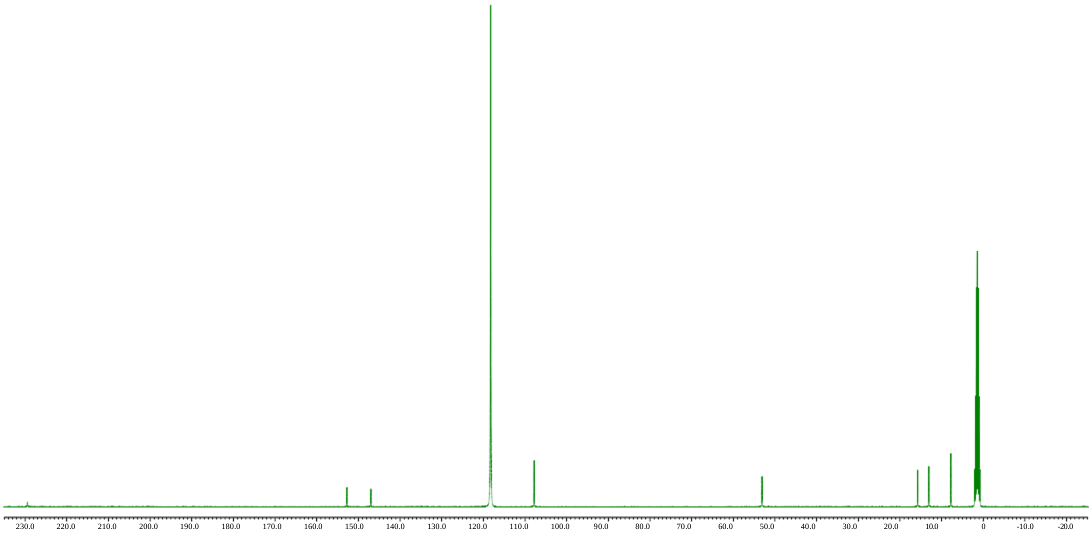

Figure S4. ^{13}C NMR spectrum (100.4 MHz, CD_3CN, 20 °C) of **2**.

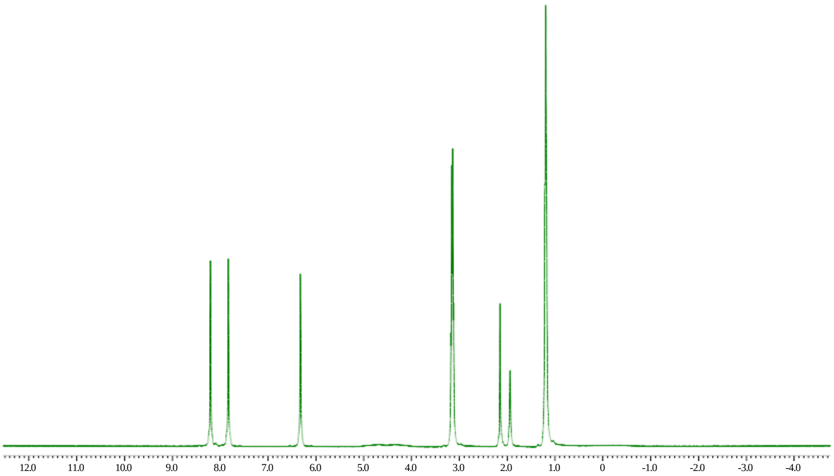

Figure S5. ^{1}H NMR spectrum (400 MHz, CD_3CN, 20 °C) of **3**.

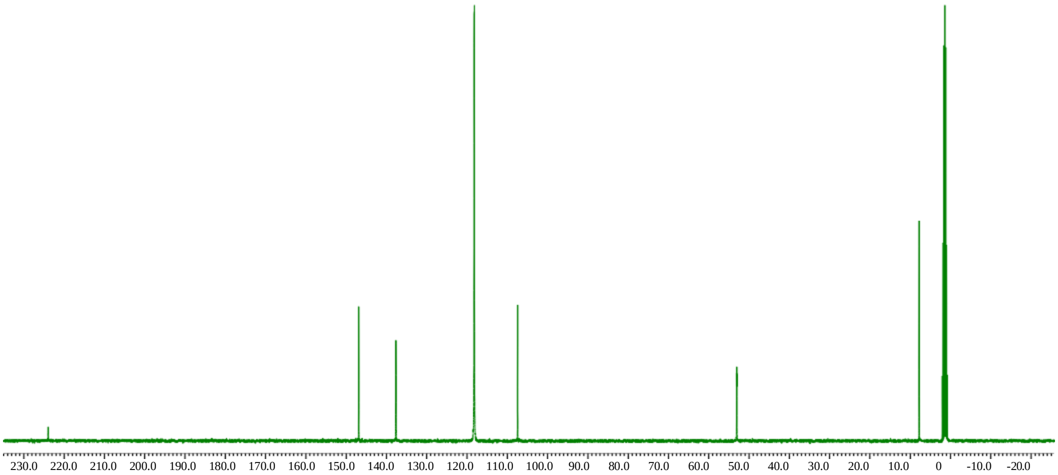

Figure S6. ^{13}C NMR spectrum (100.4 MHz, CD_3CN, 20 °C) of **3**.

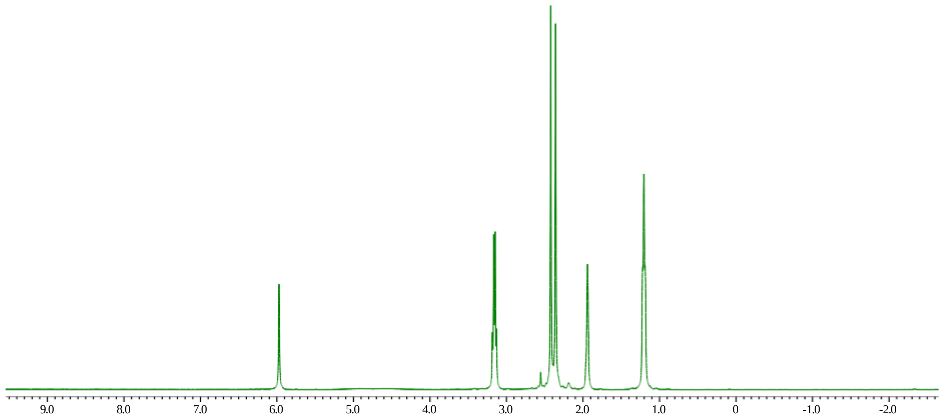

Figure S7. ^{1}H NMR spectrum (400 MHz, CD_3CN, 20 °C) of **4**.

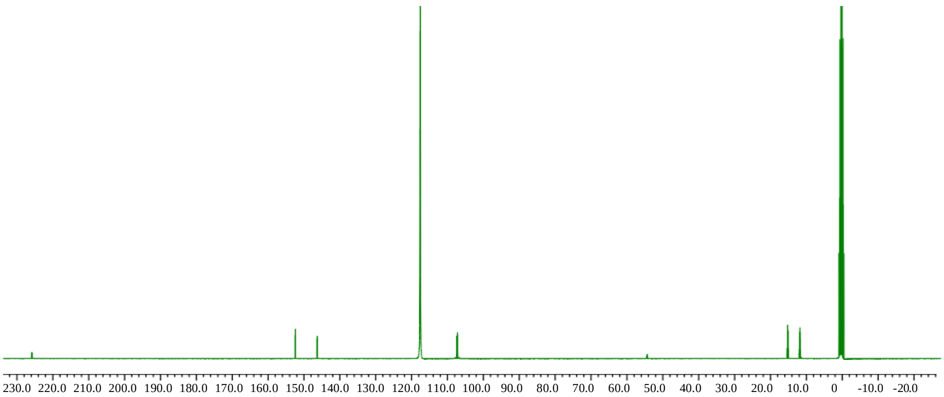

Figure S8. ^{13}C NMR spectrum (100.4 MHz, CD_3CN, 20 °C) of **4**.

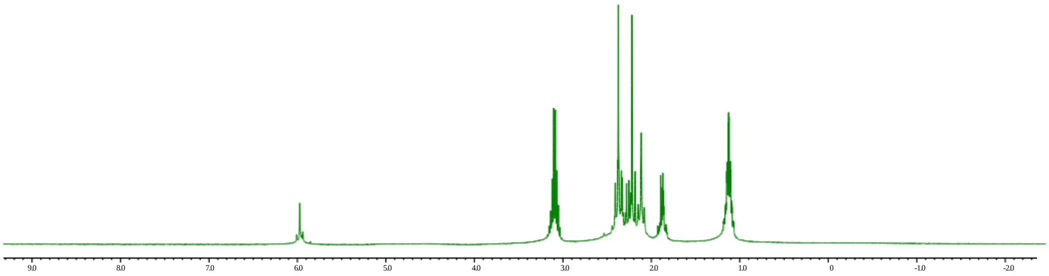

Figure S9. ^{1}H NMR spectrum (400 MHz, CD_3CN, 20 °C) of **5**.

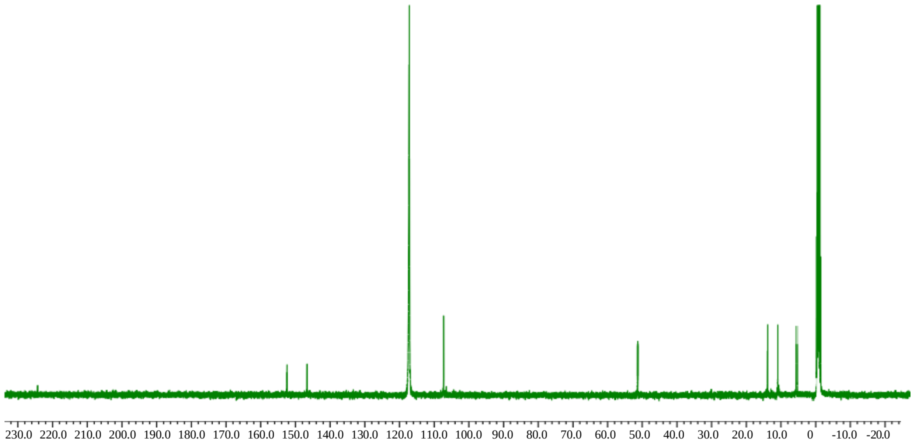

Figure S10. ^{13}C NMR spectrum (100.4 MHz, CD_3CN, 20 °C) of **5**.

Crystal packing of **1**-**5** and $Et_4N[Tp^*W(CO)_3]$

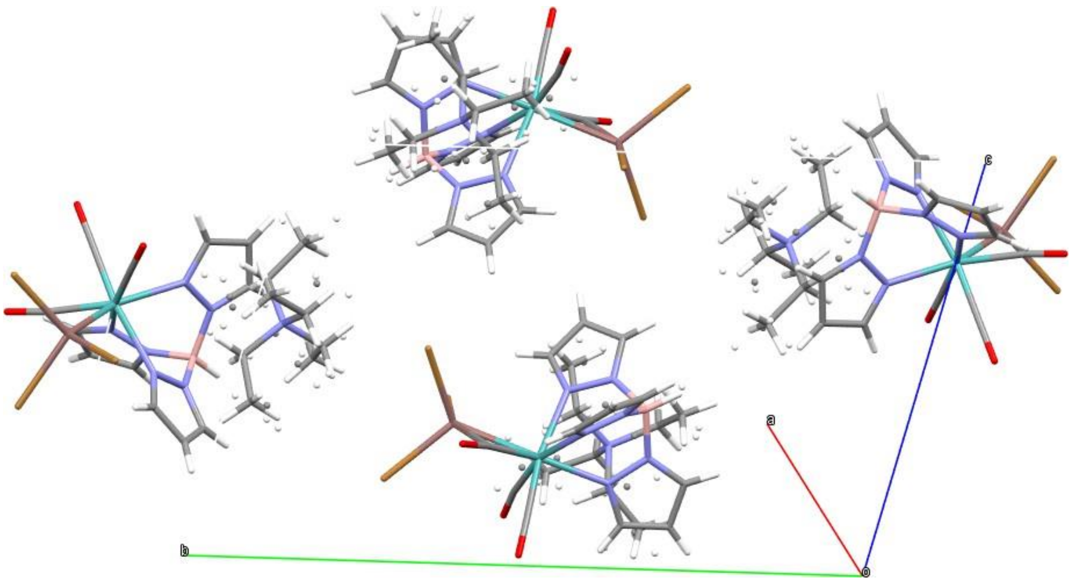

Figure S11. Crystal packing of **1**.

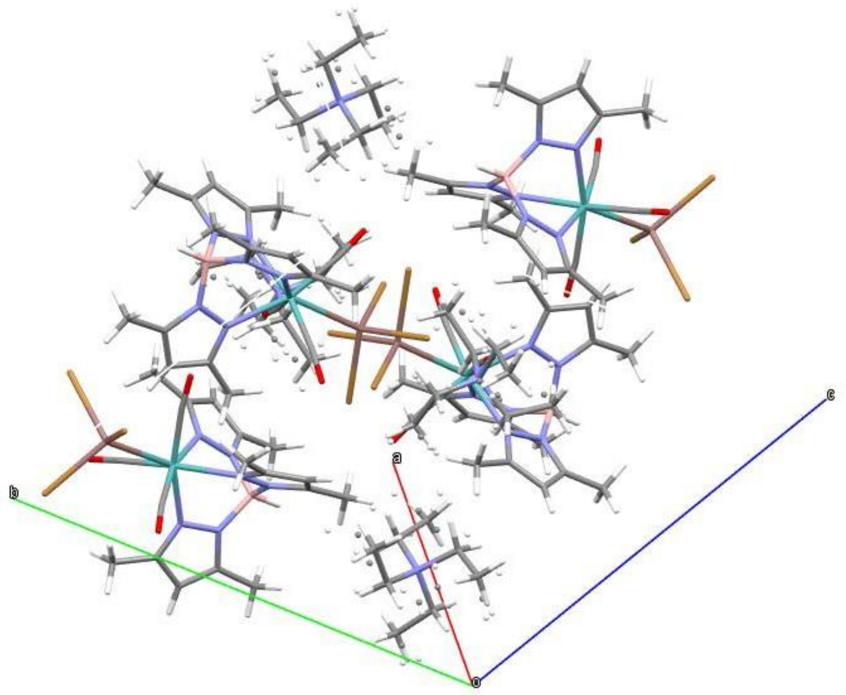

Figure S12. Crystal packing of **2**.

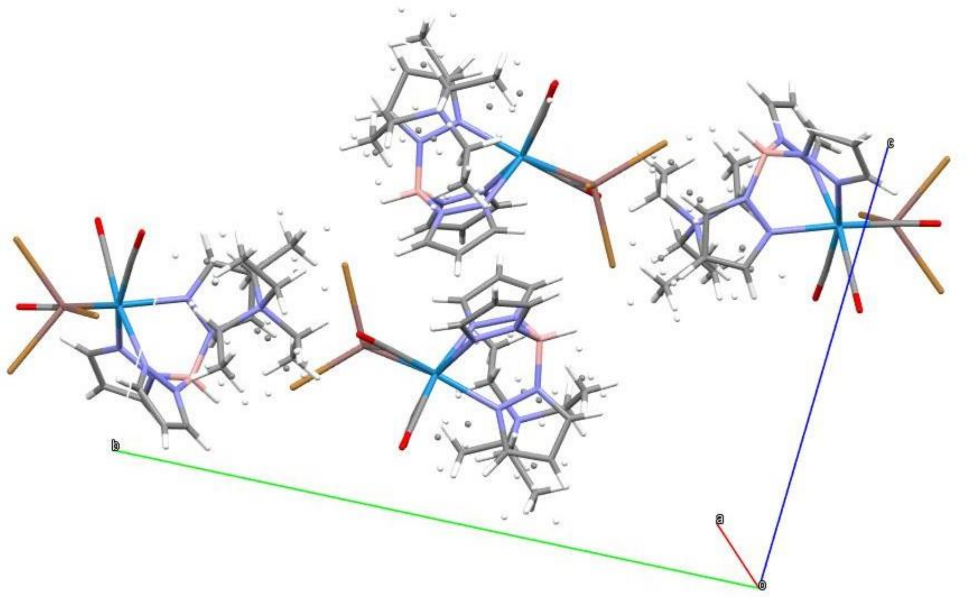

Figure S13. Crystal packing of **3**.

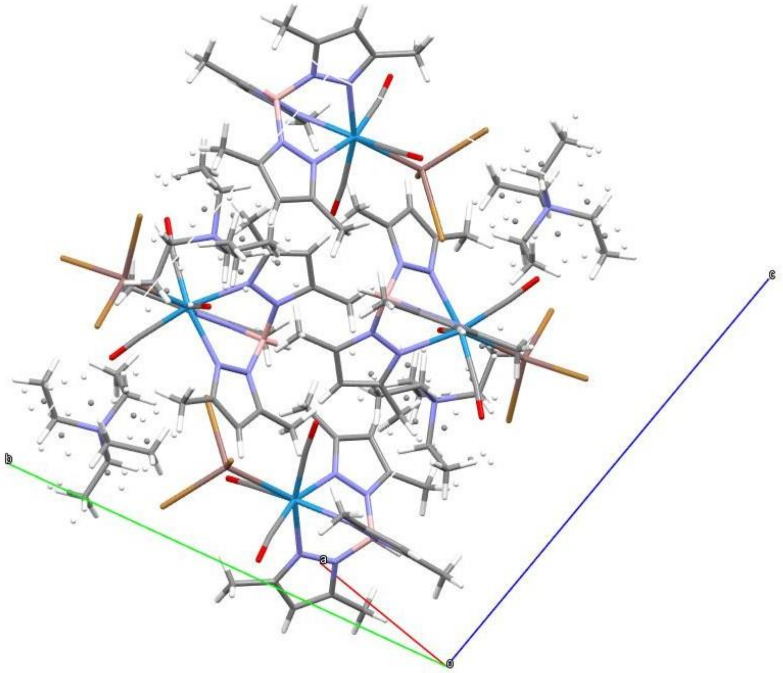

Figure S14. Crystal packing of **4**.

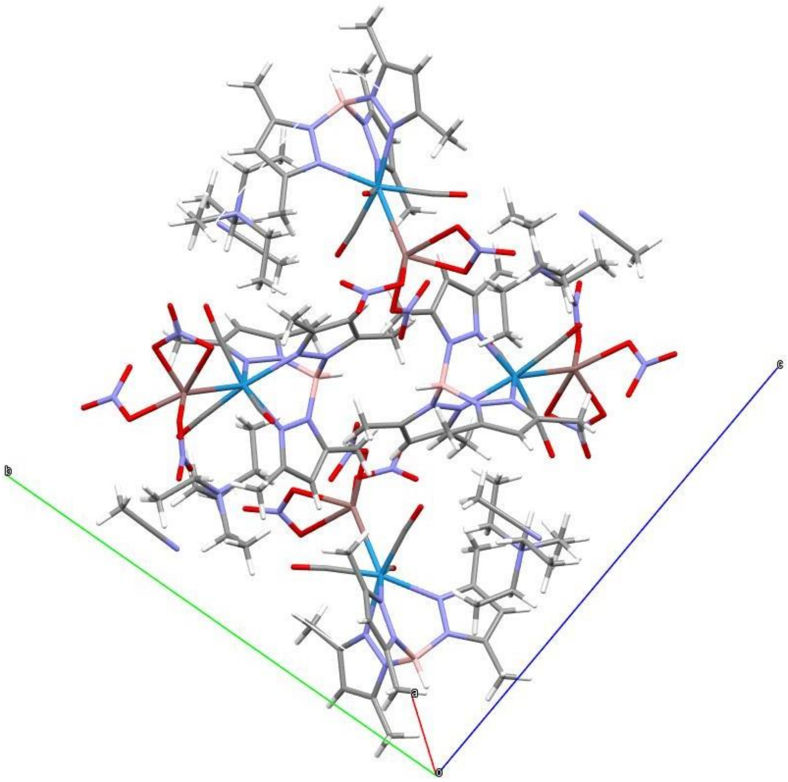

Figure S15. Crystal packing of **5**.

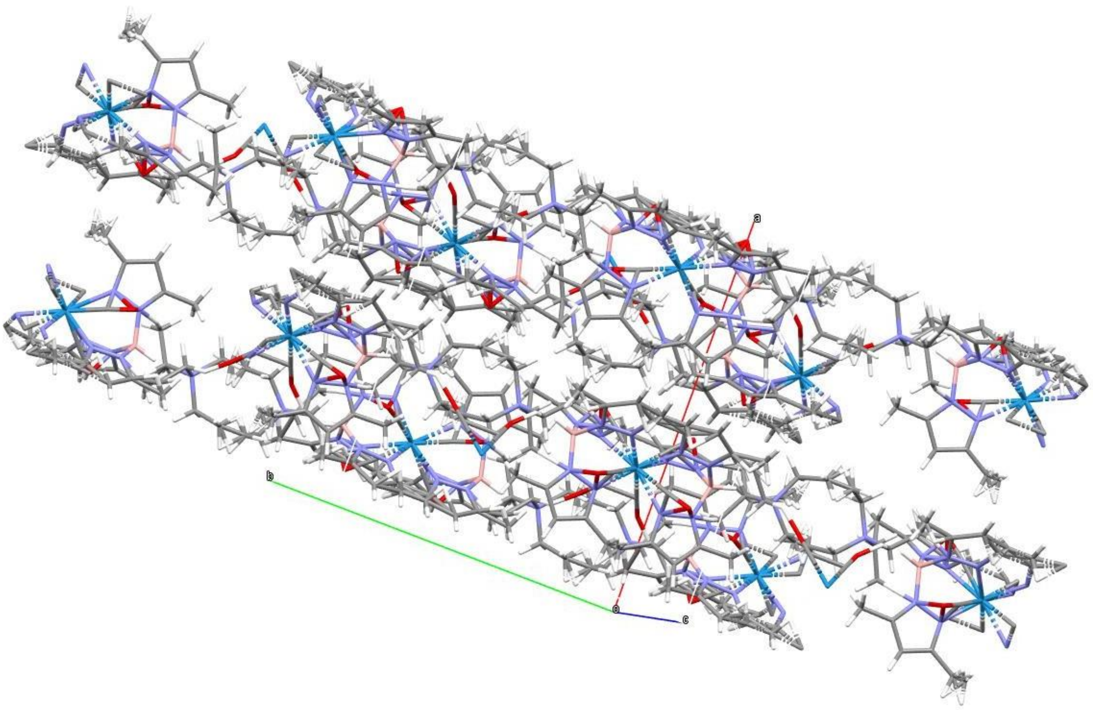

Figure S16. Crystal packing of **6**.
